# Neurobiological correlation between attention-deficit/hyperactivity disorder and obesity

**DOI:** 10.1192/j.eurpsy.2021.638

**Published:** 2021-08-13

**Authors:** A.M. Romão Franco, I. Cruz Da Fonseca, N. Ribeiro, V. Vila Nova, A. Gamito

**Affiliations:** Psiquiatria E Saúde Mental, Centro Hospitalar de Setúbal, Setubal, Portugal

**Keywords:** Dopamine, Neurobiology, ADHD, obesity

## Abstract

**Introduction:**

Attention-Deficit/Hyperactivity Disorder (ADHD) and Obesity are frequently comorbid. The prevalence of ADHD rises from around 2.8% in the general population (adults) to about 27% among those with obesity. Although neurobiological mechanisms explaining the strong association between ADHD and obesity are still unclear, several hypotheses have been proposed to explain the high comorbidity, including common genes, dopaminergic neurotransmission, deficits in executive functions (planning, adherence to weight loss programs or protocols after bariatric surgery) and circadian rhythm dysregulation.

**Objectives:**

Review on the relationship between ADHD and Obesity, focusing on possible biological mechanisms driving their high comorbidity.

**Methods:**

We conducted a search in PubMed and ClinicalKey with the terms: “Attention-Deficit/Hyperactivity Disorder”, “Obesity”, “Dopamine”.

**Results:**

Altered reward processing and impaired inhibitory control are key features of ADHD and are also related to obesity. The ability to resist the impulse to eat and an appropriate reward response require normal function of these dopamine circuits. Both ADHD and obesity are usually associated with reduced volume of putamen, known to be a fundamental player in inhibitory control functioning. Human and animal studies have also demonstrated that obese individuals have decreased dopamine D2 receptor availability in the striatum. Recently genetic analyses implicated specifically Dopamine-DARPP32 Feedback in cAMP Signaling in both ADHD and Obesity.

**Conclusions:**

ADHD and obesity are often comorbid. Dysregulated dopaminergic neurotransmission seems to be a fundamental factor underlying the overlap between ADHD and obesity, probably involving DARPP-32 signaling and possibly through neurobiological features of putamen, namely inhibitory control. Further studies are necessary to explain the neurobiological correlation between these entities.

